# Chronic Abdominal Pain After Roux-en-Y Gastric Bypass Surgery: A Prospective Cohort Study with 2-Year Follow-Up in a Swedish Population

**DOI:** 10.1007/s11695-025-08272-y

**Published:** 2025-10-21

**Authors:** Angelos Al Nimer, Hanna de la Croix, Monika Fagevik Olsén, Anna Elmered, Eva Haglind, Lars Fändriks, Eva Angenete, Hans Axelsson, Srdjan Kostic, Ville Wallenius

**Affiliations:** 1 GastroIntestinal Metabolism Science lab (GIMS), Department of Surgery, Institute of Clinical Sciences, Sahlgrenska Academy, University of Gothenburg, Gothenburg, Sweden; 2 Department of Surgery, Sahlgrenska University Hospital, Region Västra Götaland, Gothenburg, Sweden; 3 Department of Surgery, Institute of Clinical Sciences, Sahlgrenska Academy, University of Gothenburg, SSORG – Scandinavian Surgical Outcomes Research Group, Gothenburg, Sweden; 4 Department of Neuroscience and Physiology/Health and rehabilitation, Institution, Sahlgrenska Academy, Gothenburg, Sweden; 5 Department of Surgery, Skaraborgs Hospital, Skövde, Sweden

**Keywords:** Chronic abdominal pain, Physical activity, Preoperative, Obesity, Roux-en-Y gastric bypass, Gastrointestinal symptoms, Depression, Anxiety, Quality of life

## Abstract

**Background:**

The risk of developing chronic abdominal pain after Roux-en-Y gastric bypass (RYGB) surgery has come under scrutiny. Few prospective studies exist on this subject. The aim of this prospective paired analysis cohort study was to evaluate the risk of developing chronic abdominal pain and QoL 2 years after RYGB surgery.

**Methods:**

An unselected cohort of 107 patients living with obesity, scheduled to undergo elective RYGB surgery, filled out the study questionnaires before surgery. Two years after surgery, 84 patients responded to the questionnaires, resulting in a response rate of 78.5%.

**Results:**

The gastrointestinal symptom rating scale showed no change for diarrhea, indigestion, or obstipation, but on the other hand, a tendency to increased abdominal pain (*p* = 0.05) 2 years after RYGB. Gastroesophageal reflux symptoms decreased (2.1 ± 1.3 to 1.4 ± 0.9; *p* < 0.0001). Pain anxiety using the Pain Catastrophizing Scale decreased (from 13.5 ± 11.3 preoperatively to 10.1 ± 9.4 postoperatively, *p* = 0.001). The Hospital Anxiety and Depression Scale showed a decreased score for depression (4.4 ± 3.7 to 2.4 ± 3.3, *p* < 0.0001), but no change for anxiety. Quality of life increased significantly (EQ5D-3L health state from 0.69 ± 0.25 to 0.83 ± 0.23, *p* < 0.0001; EQ VAS: from 57.4 ± 19.6 to 80.1 ± 16.3, *p* < 0.001). Self-reported physical activity, according to the Saltin-Grimby Physical Activity Level Scale (SGPALS), increased (preoperative: median = 2, Q1 = 1, Q3 = 2; postoperative: median = 2, Q1 = 2, Q3 = 2.75, *p* < 0.0001).

**Conclusions:**

Our study indicates no significant increase in abdominal pain but decreased pain anxiety 2 years after RYGB surgery. Self-reported physical activity, depression symptoms, and general quality-of-life were improved compared to baseline values.

## Introduction

According to the World Health Organization, there were 890 million adults with obesity in 2022, while the worldwide prevalence of obesity has more than doubled since 1990 [1]. This makes obesity a growing challenge with its associated risk of co-morbidity [2]. Bariatric surgery is the only treatment shown to provide sustained weight reduction, decreased morbidity, and reduced mortality rate over time [3]. However, comprehensive long-term follow-up and studies on the occurrence of long-term complications such as abdominal pain are still scarce. With increasing numbers of patients being operated worldwide, some of the adverse effects of bariatric surgery have become growing entities, e.g., patients with chronic abdominal pain (CAP), that sometimes cannot be explained by well-known complications to Roux-en-Y gastric bypass (RYGB), such as gall-stone disease, stomal ulcers, internal herniations, and obstruction of the jejuno-jejunostomy. One study has indicated that patients who have undergone RYGB had higher abdominal pain scores 2–4 years after surgery compared to patients who underwent sleeve gastrectomy (SG) [4]. One of three patients with chronic abdominal pain after RYGB reported severe interference with work and daily activities [5]. Only a few observational studies, and to our knowledge no prospective studies, on chronic abdominal pain after RYGB have been published [6–9].

The aim of this study was to prospectively evaluate the occurrence of chronic abdominal pain 2 years after surgery in a random sample of patients undergoing RYGB. This study provides new insights by using a prospective design with paired pre- and postoperative data from an unselected cohort. Unlike previous research that focused only on patients with pain or used cross-sectional methods, our approach allows for within-subject comparisons over time. This design captures the development of abdominal pain more accurately and improves the generalizability and validity of our findings across the broader bariatric population.

## Methods

### Population and Setting

A prospective observational cohort study was conducted on patients who were scheduled to undergo RYGB for morbid obesity, between April 2014 and September 2015, at three participating hospitals in Sweden: Sahlgrenska University Hospital/Sahlgrenska, Sahlgrenska University Hospital/Östra, and Skaraborgs Hospital. Inclusion criteria were the same as the indications for bariatric surgery in the Western Region of Sweden: BMI > 35 kg/m^2^ with associated risk factors (diabetes, hypertension) or BMI > 40 kg/m^2^, ability to understand information and to sign an informed consent. Exclusion criteria were the inability to understand information due to language barriers, and patients who were operated on with other bariatric surgery techniques than RYGB. After giving informed consent, patients were asked to answer questionnaires (q) before surgery and 2 years after surgery. The questionnaires contained questions on quality of life (QoL), gastrointestinal symptoms, anxiety and depressive symptoms, the level of physical activity, other lifestyle factors such as smoking history and alcohol consumption, and comorbidities. A chart review was performed to reveal if there was an identifiable reason for the abdominal pain at the time the questionnaire was filled.

The study was approved by the Regional Ethical Review Authority of Gothenburg (Dnr: 310-13) and informed consent was obtained from all participants. The protocol was registered at clinicaltrials.gov with identifier NCT01707121.

### Surgical Procedure

In all patients, laparoscopic RYGB was performed with an approximately 30 mL gastric pouch, a 120 cm antecolic, antegastric alimentary limb, and a 60 cm biliopancreatic limb. The mesenteric defects were routinely closed by suturing or stapling.

### Questionnaires

A research-nurse contacted all patients before sending the pre- and post-operative questionnaires. Patients who did not return the questionnaires were reminded by one or two phone calls from the research-nurse. Questions on age, weight, and length, comorbidities (hypertension, diabetes, and hyperlipidemia), smoking, and alcohol use were followed by more specific questionnaires, namely: Gastrointestinal Symptom Rating Scale (GSRS), Saltin-Grimby Physical Activity Level Scale (SGPALS), Pain Catastrophizing Scale (PCS), Hospital Anxiety and Depression Scale (HADS), and EuroQol 5-dimension 3-Level (EQ5D-3L). All the questionnaires were validated Swedish versions, face validated, and self-administered.

The GSRS was used to evaluate the gastrointestinal symptoms and consists of 15 items organized in 5 dimensions (diarrhea, indigestion, constipation, abdominal pain, and gastroesophageal reflux). Patients responded through a 7-point Likert-type scale (1 = no discomfort and 7 = severe discomfort) [10]. A GSRS abdominal pain domain score of ≥ 3 was used to define significant abdominal pain.

A follow-up period of 2 years was chosen to comprehensively assess both short-term and long-term postoperative abdominal pain outcomes. This duration allows for the acute pain resolution, as well as the identification and characterization of chronic abdominal pain, which is typically defined as pain persisting beyond 3 months after surgery. Additionally, a 2-year follow-up captures late-onset complications such as adhesions, incisional hernias, and complications related to RYGB. For patients undergoing RYGB, this timeframe is particularly relevant, as most individuals reach their weight nadir approximately 18 to 24 months postoperatively[11]. This follow-up period also aligns with common clinical practices and enables a more accurate assessment of the long-term impact of postoperative pain on patients’ quality of life and functional recovery.

Physical activity was assessed using the 4-level SGPALS [12], known to be associated with cardiovascular risk factors [13, 14], morbidity as well as mortality [12, 15]. Patients answered the following question referring to the last week: “How much do you move and exert yourself physically during leisure time?” There were four answering categories: 1 = sedentary, 2 = some physical activity at least 4 h/week, 3 = regular physical activity/training, and 4 = regular hard physical training for competition sport.

PCS measures pain catastrophizing by assessing three components: catastrophizing rumination (“I cannot stop thinking about how much it hurts”); magnification (“I worry that something serious may happen”); and helplessness (“there is nothing I can do to reduce the intensity of the pain”) [16]. PCS is a 13-item self-reported measure of catastrophizing in the context of actual or anticipated pain, scored from 0 to 4, with a total possible score of 52. A total PCS score of 30 represents a clinically relevant level of pain catastrophizing [17].

The Hospital Anxiety and Depression Scale (HAD) is a psychometric tool that helps to assess individuals with somatic illnesses [18]. It consists of two 7-item subscales, the HADS-A (anxiety) (odd items) and the HADS-D (depression) (even items) and intends to measure the levels of anxiety and depression, respectively. Each item has a 4-point scale of frequency ranging from 0 to 3. Regarding HADS-A, patients scoring between 0 and 6 are classified as having no anxiety, though patients scoring 7 to 10 have mild anxiety, and those that have more than 10 have anxiety disorder. Patients who score between 0 and 6 on HADS-D are classified as having no depression, 7 to 10 as mild depression, and more than 10 as severe depression.

QoL was measured using the EQ5D-3L health state and the EQ Visual Analogue Scale (VAS; 0–100) [19, 20].

### Statistical Analysis

To estimate the required sample size for detecting a clinically relevant increase in abdominal pain following gastric bypass surgery, we performed a power calculation based on a paired binary outcome. Abdominal pain was assessed using the Gastrointestinal Symptom Rating Scale (GSRS), with a score of ≥ 3 (on a 1–7 scale) considered to represent clinically significant pain. Based on prior observations, we assumed that 25% of patients experience significant abdominal pain preoperatively. A 20% absolute increase in this proportion (from 25 to 45%) postoperatively was considered clinically meaningful.

Using McNemar’s test for paired binary data, and assuming a two-sided significance level of 0.05 and 80% power, we estimated that approximately 69 patients would be needed to detect this difference. The calculation assumed a conservative discordant pair proportion of 35%, representing patients who change status from below to above the GSRS pain threshold or vice versa. We chose to include 107 patients to strengthen the robustness and generalizability of our findings and to account for potential drop-outs and loss to follow-up.

All statistical analyses were made using Excel and GraphPad Prism 6 statistics software and SPSS Statistics 27 software. All analyses were performed in a paired fashion using Student’s t-test with Bonferroni correction for multiple analyses or Wilcoxon test with Dunn’s test for multiple comparisons and chi-square. Correlations were tested using the Spearman coefficient test. Change of the GSRS, HAD-anxiety, HAD-depression, and PCS score were defined as the postoperative score minus the score at baseline [10, 17, 18, 21]. *p* < 0.05 was considered statistically significant. *r* < 0.3 was considered a significant correlation.

This manuscript was prepared in accordance with the STROBE (Strengthening the Reporting of Observational Studies in Epidemiology) guidelines for observational studies.

## Results

**Fig. 1:**
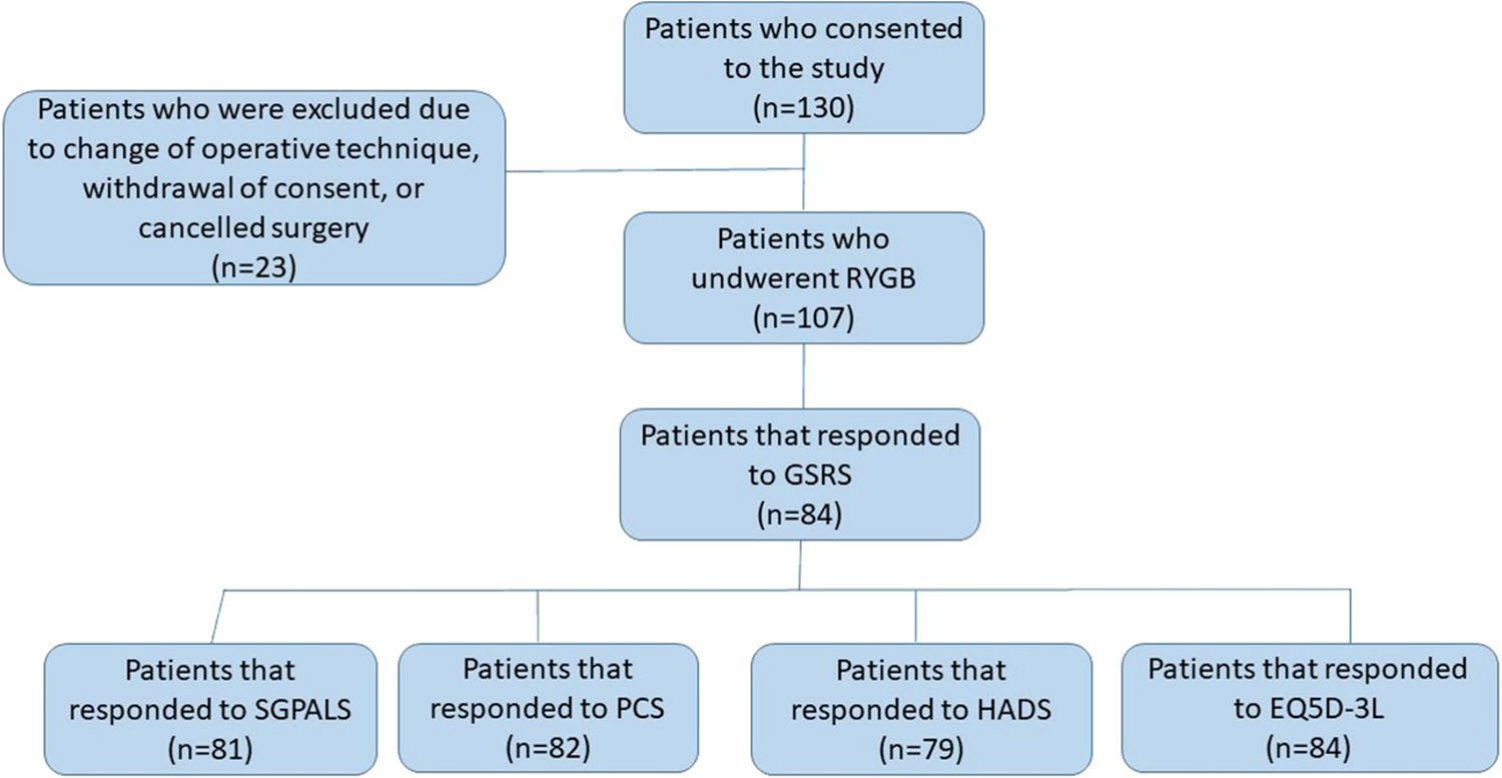
Schematic illustration of the study design. The bottom squares show how many patients responded to the questionnaires both before and after surgery. These figures represent the same individuals who provided responses at both time points, ensuring paired pre- and postoperative data for analysis. GSRS = Gastrointestinal Symptoms Rating Scale; SGPALS = Saltin-Grimby Physical Activity Level Scale; PCS = Pain Catastrophizing Scale; HADS = Hospital Anxiety and Depression Scale

A total of 130 patients consented to participate in the study. Twenty-three patients were excluded due to a change of operative technique, cancelled surgery, or withdrawal of consent. Of the remaining 107 patients, 84 (78.5%) responded to the Gastrointestinal Symptom Rating Scale questionnaire (GSRS) prior to and 2 years after surgery. Excluded patients and response rates to the questionnaires are shown in Fig. 1. Age, sex, smoking habits, BMI, and obesity-related diseases, before and 2 years after surgery, are shown in Table 1.

**Table 1:** Patient characteristics at baseline and 2 years after Roux en Y gastric bypass

Patient characteristics	Before surgery	After surgery
Number of patients	84	84
Age (years): mean (SD)	43.3 (SD12.1)	45.3 (12.1)
Female/male: *n* (%)	65/19 (77/23)	65/19 (77/23)
Weight (kg): mean (SD)	126 (20)	83.3 (15)
BMI (kg/m^2^): mean (SD)	43.4 (5)	28.6 (4)
Diabetes: yes/no (%)	16/68 (19/81)	10/74 (12/88)
Hypertension: yes/no (%)	28/56 (33/67)	18/66 (21/79)
Hyperlipidemia: yes/no (%)	11/73 (13/87)	5/79 (6/94)
Current smoker: yes/no (%)	18/66 (21/79)	18/66 (21/79)
Alcohol overconsumption (AUDIT): yes/no (%)	6/78 (7/93)	10/74 (12/88)

*n* number, *SD* standard deviation

The average weight and BMI decreased after 2 years to an expected level of 34% (weight from 126.0 ± 20.0 to 83.3 ± 15.0 kg; mean ± SD, *p* < 0.0001; BMI from 43.4 ± 5 to 28.6 ± 4 kg/m^2^; *p* < 0.0001). The proportion of patients with obesity-related diseases such as hypertension, diabetes, and hyperlipidemia decreased one year after surgery, while their smoking and alcohol habits did not change (Table 1).

### Gastrointestinal Symptoms According to the Gastrointestinal Symptom Rating Scale

**Fig. 2:**
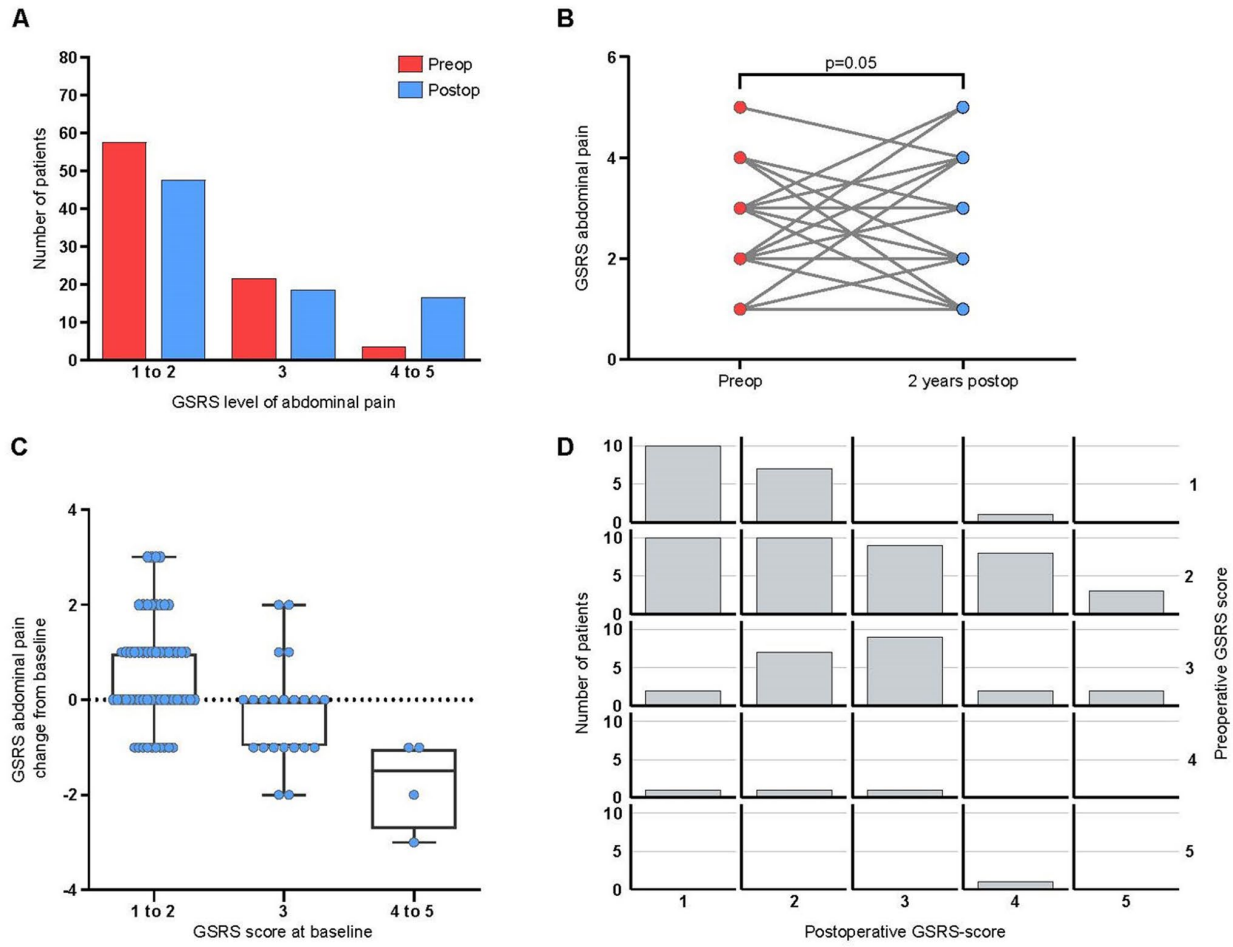
GSRS levels of abdominal pain on 84 patients at baseline and 2 years after Roux en Y gastric bypass. **A** Number of patients with abdominal pain GSRS level 1 to 2, GSRS level 3 and GSRS 4 to 5 at baseline and 2 years after Roux en Y gastric bypass. **B** Change of GSRS abdominal pain level before and 2 years after surgery. **C** Change of 2-years GSRS abdominal pain level in relation to baseline. **D** Distribution of GSRS pain score at 2 years postop in relation to pain score at baseline

The number of patients that reported no or minor discomfort (GSRS abdominal pain score 1 to 2), mild discomfort (GSRS abdominal pain score 3), and moderate or moderately severe discomfort (GSRS abdominal pain score 4 to 5) before and 2 years after surgery are shown in Fig. 2a. A tendency to increased average score in the whole group of patients for abdominal pain 2 years after RYGB was observed (average for whole group from 2.1 ± 0.8 to 2.4 ± 1.2; mean ± SD; unadjusted non-parametric paired analysis *p* = 0.05) (Fig. 2b). Thirty-two of 84 patients (38.1%) reported an increased GSRS score for abdominal pain after surgery, with a mean increase from 1.9 ± 0.6 to 3.4 ± 1.0. Twenty-three reported decreased pain (27.4%) with a mean decrease from 2.8 ± 0.9 to 1.6 ± 0.8, and 29 (34.5%) reported no change.

Looking more in detail at the 26 patients (30.1%) that reported a GSRS abdominal pain score of three or higher before surgery, indicating preexisting “significant” abdominal pain, only four (15.4%) reported an increased score postoperatively after RYGB, while 13 (50%) reported a decreased score and nine (34.6%) patients reported no change (Fig. 2b). Indeed, when stratifying the patients into three different groups according to the severity of the discomfort at baseline, those with no or minor discomfort reported an increase in abdominal pain after surgery, while those with mild and severe discomfort reported a decrease (Fig. 2c and in detail in Fig. 2 d). However, of the 32 patients who reported an increase in abdominal pain 2 years after surgery, 25 patients (29.7%) had a GSRS score of 3 or more, indicating significant abdominal pain. An identifiable reason, after chart review, was found in seven of the 36 patients that reported chronic abdominal pain 2 years after surgery. One patient was reoperated with jejunojejunostomy reconstruction due to invagination and later bowel obstruction, two other patients were operated on due to bowel obstruction caused by adhesions, one patient had a cholecystectomy and was reoperated because of bile leakage, one patient was found to have an ulcer in the gastroenteroanastomosis, and two patients were operated on due to internal herniation.

The GSRS showed no change for diarrhea, indigestion, or obstipation. A significant decrease of gastroesophageal reflux symptoms was observed (2.1 ± 1.3 to 1.4 ± 0.9; *p* < 0.001).

### Physical Activity

Self-reported PA according to the SGPALS was increased in 34 of 81 patients 2 years after surgery, decreased in four, and unchanged in 43 patients. Twenty-six patients changed their PA level from physically inactive to active 2 years postoperatively (from category one to two or higher). Two years after surgery, 83% were physically active in contrast to 52% before surgery, now belonging to categories two (some physical activity at least 4 h/week; 57%), three (regular physical activity/training; 21%), or four (regular hard physical training for competition sports; 5%).

### Mental Health Measurements

Pain anxiety, measured using the PCS, decreased from baseline to 2 years (13.5 ± 11.3 vs. 10.1 ± 9.4, *p* = 0.001). The HADS showed a lower score for depression (4.4 ± 3.7 to 2.5 ± 3.3, *p* < 0.0001) but stable levels for anxiety at 2 years after surgery.

### Quality of Life

Both EQ5D-3L health state QoL, as well as EQ VAS QoL, were significantly improved after 2 years (EQ5D-3L health state from 0.69 ± 0.25 to 0.83 ± 0.23, *p* < 0.0001; EQ VAS: from 57.4 ± 19.6 to 80.1 ± 16.3, *p* < 0.001).

### Correlations

There was a positive correlation of the GSRS score and the HAD-A score both at baseline and 2 years after surgery (Table 2). Similarly, the GSRS score correlated positively with the HAD-D score 2 years after surgery, and there was a tendency for a correlation also at baseline. In addition, an increase in the GSRS score after surgery was associated with an increase in the HAD-A score. On the other hand, there was no association between the change in GSRS score and the change in HAD-D score. All three associations between the GSRS score and the PCS score, i.e., at baseline, 2 years after surgery, and the change, were significant (Table 2).

**Table 2:** Association between GSRS score and HAD-anxiety, HAD-depression, and PCS score at baseline, 2 years after surgery as well as the change in between

Tested associations (Spearman)	Spearman *r*	*p* value
GSRS score vs HAD-A: baseline	0.41	0.0002
GSRS score vs HAD-A: 2y after surgery	0.56	< 0.0001
GSRS score vs HAD-A: change	0.34	0.0024
GSRS score vs HAD-D: baseline	0.22	0.0506
GSRS score vs HAD-D: 2y after surgery	0.38	0.0006
GSRS score vs HAD-D: change	0.02	0.8527
GSRS score vs PCS score: baseline	0.45	< 0.0001
GSRS score vs PCS score: 2y after surgery	0.50	< 0.0001
GSRS score vs PCS score: change	0.28	0.0105

*GSRS* gastrointestinal symptom rating scale, *HAD-A* Hospital Anxiety and Depression Scale-anxiety, *HAD-D* hospital anxiety and depression scale-depression, *PCS* pain catastrophizing scale, 2y 2 years after surgery

## Discussion

In this prospective cohort study, we did not find that the overall score of abdominal pain in the randomly selected subjects with obesity undergoing RYGB changed. Before surgery, 31% of patients reported a GSRS abdominal pain score ≥ 3, and 2 years after surgery, this number was 43% of patients. This is similar to earlier reports at five years after surgery [6]. Of the 36 patients that reported chronic abdominal pain 2 years after surgery, an identifiable reason was found in 19% upon chart review at the time of the inquiry. Thus, the number of patients with GSRS ≥ 3 without an identified reason for the abdominal pain was 35% 2 years after surgery compared to 31% before surgery. This number, along with the 42% of patients with postoperative GSRS ≥ 3 who also reported preexisting preoperative CAP at the same level, indicates significantly lower postoperative onset of chronic abdominal pain compared to an earlier study [6]. These differences may depend on the study setup, with differences in patient selection. In our study, all patients operated on were studied and not only those who reported abdominal pain after surgery, as in the aforementioned study [6].

In patients who develop chronic abdominal pain following RYGB without identifiable anatomical or surgical complications, functional gastrointestinal disorders (FGIDs) such as visceral hypersensitivity or functional abdominal pain syndromes may be important underlying contributors. These conditions, including disorders like irritable bowel syndrome (IBS), are characterized by altered gut-brain interaction, central sensitization, and heightened perception of visceral stimuli, often in the absence of clear structural pathology. Bariatric surgery itself can induce significant changes in gastrointestinal motility, neurohormonal signaling, and gut microbiota, which may predispose some individuals to functional pain syndromes [22]. Our baseline findings with a high proportion of patients with gastrointestinal symptoms are in line with the Rome Foundation Global Study where 40% of the studied population met the criteria for at least one of the syndromes that are included in the functional gastrointestinal disorders [23]. In another study, RYGB patients displayed 29% chronic abdominal pain 2 years after surgery, vs. 28% in sleeve gastrectomy patients [5]. Yet another study reported self-reported CAP after RYGB in 39% of subjects compared to 11% seeking medical care for abdominal pain in a reference population. The total frequency of CAP in the reference populations was reportedly up to 20% [8]. In our present study, patients reporting a GSRS abdominal pain score of ≥ 4 corresponding to at least moderate abdominal pain were 3/84 patients (4%) before surgery and 17/84 patients (20%) 2 years after surgery. Of these 17 patients with postoperative GSRS ≥ 4, six patients displayed an identified diagnosis, resulting in 11 patients (13%) remaining with unexplained CAP. Thus, this group of patients with more severe abdominal pain without an identified diagnosis was significantly increased (*p* = 0.026). Another interesting finding was that the four patients reporting the highest GSRS abdominal score (4 and 5) before surgery reported decreased GSRS scores after surgery. Of these 17 patients with the highest GSRS abdominal score 2 years after surgery, 12 were found among patients that before surgery reported low or no abdominal pain score (GSRS abdominal pain ≤ 2), four with GSRS score of 3 and one who reported 5. Thus, preexisting abdominal pain before RYGB does not seem to be a good selector, i.e., exclusion criterion for patients to receive RYGB surgery. There was a significant correlation between abdominal pain and HAD-D, HAD-A, and PCS, in line with an earlier study [7], but more studies are needed to investigate the causal relationship of these associations.

A strength of this study was the paired-analysis prospective design and that compliance was high, 78.5% with 84/107 patients completing the postoperative questionnaires. Weaknesses included the low number of participants, the duration of follow-up, and the difficulty of defining chronic abdominal pain. Although the initial sample size exceeded the minimum required by our power analysis, the final number of patients who completed follow-up (*n* = 84) was relatively small, which may limit the ability to detect smaller effects and perform robust subgroup analyses. While the two-year follow-up provides meaningful insight into the development of chronic abdominal pain after RYGB, it is possible that some complications or changes may manifest beyond this time frame. Another limitation is that we did not collect postoperative variables such as medication use, adherence to dietary and physical activity recommendations, and other health-related changes, which limits our ability to assess their potential influence on symptoms and quality of life. Future studies with larger sample sizes, longer follow-up, and the study of the mentioned postoperative variables are needed to confirm and extend these findings. However, we used the well-established GSRS abdominal pain score for this evaluation. Patients experiencing chronic abdominal pain would seem likely to report this using the GSRS. In contrast, we may have caught patients in this group only experiencing transient abdominal pain at the time of the inquiry. This was also indicated by chart review, with some patients reporting high GSRS scores in the inquiry, but where clinical follow-up by bariatric nurses at or close in time did not reveal any such symptoms. Thus, our numbers of abdominal pain may indicate some over-estimation, rather than under-estimation of “chronic” abdominal pain. We found rather a high number of patients with obesity report preexisting abdominal pain before RYGB surgery. The total number of patients with abdominal pain in the whole cohort did not show a significant increase, but there seems to be a subgroup of patients with little or no preexisting pain that develop severe abdominal pain symptoms 2 years after surgery.

Although the threshold of GSRS ≥ 3 is not formally validated, it has been employed in previous studies as a cut-off for clinically relevant abdominal pain; therefore, we adopted the same criterion to maintain consistency and comparability with prior publications [7].

An additional limitation is that advanced diagnostic modalities such as endoscopy, imaging, or functional gastrointestinal studies were not systematically performed in this study. While some patients underwent further investigations based on clinical need, a standardized diagnostic workup might have uncovered structural or functional causes of abdominal pain not otherwise identified. Future studies including protocolized assessments could help clarify the underlying mechanisms of CAP after RYGB and also try to find predictors for patients at risk of developing severe chronic abdominal pain after RYGB.

### Clinical Implications

The findings of this study highlight that a subset of patients undergoing RYGB may develop CAP in the absence of an identifiable structural cause. This underscores the need for clinicians to monitor for pain symptoms even in patients without obvious complications and to consider FGIDs and psychological factors in the differential diagnosis. Importantly, our results suggest that preexisting abdominal pain should not be considered an exclusion criterion for patients undergoing RYGB, as pain symptoms may evolve postoperatively regardless of baseline status, and a number of patients with preexisting abdominal pain actually experience a decrease in pain following surgery. Early recognition of CAP and its potential impact on quality of life may prompt timely referral to multidisciplinary care teams, including gastroenterologists, psychologists, and dietitians. Identifying predictors of CAP could also help tailor preoperative counseling and postoperative follow-up, ultimately improving patient outcomes.

## Data Availability

Data underlying this article will be shared by the corresponding author upon reasonable request, in accordance with institutional and ethical guidelines.
